# Maternal hyperuricemia and adverse neonatal outcomes in normotensive pregnancies: Evidence from a prospective cohort study in Eastern Uganda

**DOI:** 10.1371/journal.pone.0342913

**Published:** 2026-03-02

**Authors:** Hassan Abdullahi Hafsa, Marie Pascaline Sabine Ishimwe, Musa Kasujja, Geoffrey Okot, Sawda Abdikarim Sheikh Isse, Hussein Mire Hamdi, Mohamud Mohamed Sadia, Ramlo Abdi Ali, Adam Abdrahim Suliman Ebaid, Maxwell Okello, Ahmed Kiswezi Kazigo, Theodore Nteziyaremye, Ibrahim Bwaga, Theoneste Hakizimana

**Affiliations:** 1 Department of Obstetrics and Gynecology, Kampala International University, Ishaka, Uganda; 2 Department of Pediatrics and Child Health, Kampala International University, Ishaka, Uganda; 3 Department of Surgery, Kampala International University, Ishaka, Uganda; 4 Department of Sciences, University of Rwanda, Kigali, Rwanda; International University of Health and Welfare, School of Medicine, JAPAN

## Abstract

**Background:**

Maternal hyperuricemia is a potential biomarker of adverse pregnancy outcomes, but evidence among normotensive women in low- and middle-income countries is limited. We aimed to assess the association between elevated maternal serum uric acid (SUA) levels and adverse neonatal outcomes.

**Methods:**

We conducted a prospective cohort study at Jinja Regional Referral Hospital, Uganda, from 1^st^ October 2024–1^st^ February 2025, enrolling 352 normotensive women in latent labor. SUA was measured using gestational-age–specific cutoffs, classifying women as hyperuricemic or normouricemic. Outcomes included preterm birth, small-for-gestational-age (SGA) birth, stillbirth, and low Apgar score. Poisson regression with robust standard errors estimated adjusted relative risks (aRR) and 95% confidence intervals.

**Results:**

Adverse neonatal outcomes occurred in 37.5% of participants. Hyperuricemic women had significantly higher composite risk (62.5% vs. 12.5%; aRR = 4.32, 95% CI 2.92–6.39). Hyperuricemia independently predicted preterm birth (aRR = 3.63, 95% CI 1.70–7.72), SGA (aRR = 2.80, 95% CI 1.61–4.86), and stillbirth (aRR = 5.00, 95% CI 1.47–16.99). Maternal age ≥ 40 years, low education, < 4 antenatal visits, previous preterm birth, previous stillbirth, and obesity were also associated with adverse outcomes. **Conclusions.**

Maternal hyperuricemia is a strong predictor of preterm birth, SGA, and stillbirth. Routine SUA screening during antenatal care, combined with closer monitoring of high-risk women, could help improve neonatal outcomes.

## Introduction

Adverse neonatal outcomes remain a major global health concern, particularly in low- and middle-income countries (LMICs), where they contribute substantially to neonatal and under five mortality with a global estimate of 2.9 million neonates who die within the first month of life [1,2]. Sub-Saharan Africa bears the highest burden accounting for 38% of all neonatal deaths with over 75% of these deaths occurring within the first week of life [3,4]. In Uganda, the neonatal mortality rate is 19 per 1,000 live births, with preterm birth, low birth weight (LBW), and neonatal infections as the leading causes [5,6]. Approximately 15–20% of all births worldwide are affected by LBW, increasing the risk of hypothermia, hypoglycemia, infections, and neonatal mortality [5,7]. Preterm birth complicates nearly 15 million pregnancies annually, with over one million neonates dying from prematurity-related complications [8]. Additionally, 2.6 million stillbirths occur globally each year, with the highest burden reported in LMICs (5). These statistics underscore the need for early risk identification and targeted interventions to reduce neonatal morbidity and mortality.

Emerging evidence suggests that maternal biochemical markers, such as serum uric acid levels, may play a role in predicting adverse neonatal outcomes. Uric acid, a byproduct of purine metabolism, has dual properties: while it acts as an antioxidant under normal physiological conditions, elevated levels are linked to oxidative stress, endothelial dysfunction, systemic inflammation, and placental insufficiency, all of which can impair fetal growth and development [9]. Hyperuricemia has been extensively studied in hypertensive pregnancies, particularly in preeclampsia and gestational hypertension, where it is associated with adverse neonatal outcomes such as intrauterine growth restriction, preterm birth, and perinatal death [10]. However, its role in normotensive pregnancies remains unclear.

Recent studies suggest that even in the absence of hypertension, maternal hyperuricemia may contribute to poor neonatal outcomes. Amini et al. found that SGA births were significantly more common in normotensive hyperuricemic women (11.3%) than in normouricemic women, and NICU admissions were also greater in the hyperuricemic group (19.5%) [11]. Jayaraj et al. reported that neonates born to hyperuricemic mothers had increased risks of low Apgar scores (<7) (82% vs. 56%), preterm birth (44% vs. 18%), and NICU admissions (58% vs. 34%) [12]. Similarly, Yuan et al, in a large retrospective cohort study of 11,580 women, reported that those in the highest quartile of serum uric acid had a significantly higher risk of LBW (AOR = 2.63, 95% CI: 1.76–3.95) and SGA (AOR = 2.11, 95% CI: 1.73–2.57) [13]. Furthermore, Laughon et al. reported that 30.8% of normotensive hyperuricemic women delivered SGA infants whereas only 3.4% of normouricemic women did (p < 0.001), suggesting that uric acid may be an independent predictor of fetal growth restriction [14].

In Uganda, a recent prospective cohort study at Mbarara Regional Referral Hospital among women with preeclampsia reported that maternal hyperuricemia (>6 mg/dL) was associated with markedly higher rates of composite adverse perinatal outcomes, including fresh stillbirth, low birth weight, low Apgar score at 5 minutes, NICU admission and early neonatal death [15] Unlike that study, which focused exclusively on hypertensive pregnancies, our cohort was restricted to normotensive women in Eastern Uganda.

Despite these findings, there is limited research on the impact of maternal hyperuricemia on neonatal outcomes in normotensive pregnancies, particularly in sub-Saharan Africa. Given the high burden of adverse neonatal outcomes in Uganda and the absence of routine uric acid screening in antenatal care, understanding this association could provide critical insights for early risk identification and intervention. This study aims to bridge this knowledge gap by assessing the relationship between maternal hyperuricemia and adverse neonatal outcomes in normotensive pregnancies, ultimately informing the potential integration of uric acid screening into antenatal care.

## Methods

### Study design and setting

This prospective cohort study was conducted in the labor ward of Jinja Regional Referral Hospital (JRRH) from 1^st^ October 2024–1^st^ February 2025. JRRH is a tertiary referral hospital that serves a large population in eastern Uganda and provides comprehensive maternal and neonatal healthcare services to districts including Buikwe, Kayunga, Kaliro, Jinja, Bugiri, Kamuli, Mayuge, Namayingo, Iganga, and Luuka. The Department of Obstetrics and Gynecology has four delivery beds and manages an average of 15–20 births daily, with a significant proportion of neonates experiencing immediate adverse outcomes (JRRH, 2023, unpublished). The department is staffed by a multidisciplinary team comprising one senior consultant, three consultants, five senior house officers, two medical officers, and 12 nurses. Additionally, the hospital has an accredited laboratory capable of performing a wide range of diagnostic tests, including hematological, biochemical, parasitological, microbiological, and serological tests.

### Study population

This study included all normotensive pregnant mothers at or beyond 28 weeks with systolic blood pressure <140 mmHg and diastolic blood pressure <90 mmHg, provided that they consented to participate. Women with multiple pregnancies, preexisting conditions affecting uric acid metabolism (chronic kidney disease, diabetes, gout) and those on medications known to alter uric acid levels (diuretics, corticosteroids, methotrexate) were excluded from the study. Women with chronic kidney disease were excluded if they had a documented diagnosis of chronic renal disease or chronic kidney failure in their antenatal records or hospital file. Women with pre-existing diabetes mellitus were excluded if they had a prior diagnosis of type 1 or type 2 diabetes, were using glucose-lowering medication (e.g., insulin or oral hypoglycemics), or had diabetes documented in their antenatal records.

### Sample size determination

We estimated the minimum required sample size using the Kelsey formula for cohort studies, assuming a two-sided α of 0.05, 80% power and an exposed: unexposed ratio of 1:1. We used proportions of low Apgar score (<7 at 5 minutes) of 17.5% among hyperuricemic mothers and 7.6% among normouricemic mothers, based on Noman et al. [16]. Under these assumptions, the required sample size was 176 women per group. Therefore, the minimum required sample size for this study was 352 normotensive women in latent labor.

### Sampling technique

A systematic sampling approach with a sampling interval of three was employed. The first participant was randomly selected from among the first ten eligible women presenting each day. Subsequently, every third eligible woman on the sampling list was approached. If a selected woman declined participation, the next woman in the sequence was invited instead. This process continued until the required daily sample size was achieved.

All participants who met the inclusion criteria and provided written informed consent were enrolled. Upon recruitment, they completed a structured questionnaire and underwent blood sampling for serum uric acid analysis.

The participants were categorized into 2 groups: The hyperuricemic group, in which maternal serum uric acid (SUA) levels ≥1 standard deviation above the gestational age-specific normal value and the normouricemic group in which the maternal serum uric acid level was < 1 standard deviation above the gestational age-specific normal value.

### Data collection procedure

All normotensive mothers in the latent phase of labor were given all the details about the study followed by informed written consent provided that they were able to make voluntary decisions. Structured interviewer-administered questionnaires in the local language or English were completed and a review of hospital patient records was performed to gather data for analysis. Blood sampling for the uric acid test was performed and the results were communicated to mothers individually and advised to link up with specialists on duty. For appropriate management. After delivery, neonatal outcomes were extracted from delivery and neonatal records.

### Study variables

The independent variables in this study included maternal sociodemographic, obstetric and medical factors associated with adverse outcomes in normotensive mothers with or without hyperuricemia.

The dependent variable was adverse neonatal outcomes recorded from the delivery room or postnatal ward referring to either preterm birth, small-for-gestational age, stillbirth or low Apgar score. Preterm birth, small-for-gestational-age (SGA), stillbirth and low Apgar score at 5 minutes were pre-specified as primary neonatal outcomes. Early neonatal death was treated as a secondary exploratory outcome

### Diagnosis of hyperuricemia

Four milliliters of venous blood were drawn into plain serum tubes during the latent phase of labor. Samples were transported at room temperature to the hospital laboratory and allowed to clot, then centrifuged within approximately 30 minutes at 3,000 rpm for 5 minutes. Serum was analysed for uric acid using the hospital’s routine enzymatic colorimetric method according to the manufacturer’s instructions (Cobas 6000, Roche Diagnostics, Germany). When immediate analysis was not feasible, serum aliquots were stored at 2–8 °C and assayed within 24 hours; no samples included in this analysis were frozen or stored beyond 24 hours prior to measurement. Hyperuricemia was defined on the basis of gestational age-adjusted thresholds, consistent with established literature [17]. A serum uric acid level ≥1 standard deviation above gestational age-specific normal values was classified as hyperuricemia:

Gestational-age–specific reference values were derived from the longitudinal curves described by Lind et al., who quantified serial changes in serum uric acid concentrations during normal pregnancy [17]. These curves are widely used as pregnancy-specific reference ranges in studies of uric acid and pregnancy outcomes and in the absence of population-specific gestational reference curves for Uganda, we therefore adopted the Lind et al. values and defined hyperuricemia as a serum uric acid concentration ≥1 standard deviation above the gestational age–specific mean, emphasizing the woman’s relative position within the pregnancy-specific distribution rather than a fixed cut-of (Table 1).

**Table 1:** Serum Uric acid levels according to Gestational age.

Gestational Age	Serum Uric Acid (mg/dL)
28–32 weeks	≥ 4.50
32 W + 1D - 33 W	≥ 4.70
33 W + 1D - 34 W	≥ 4.93
34 W + 1D - 35 W	≥ 4.98
35 W + 1D - 36 W	≥ 5.04
36 W + 1D - 37 W	≥ 5.40
38 weeks and over	≥ 5.58

### Quality control

To ensure data accuracy and reliability, quality control measures were implemented at both the data collection and laboratory processing stages. During questionnaire administration, the researcher ensured that all the questions were presented in simple, clear language, minimizing the number of technical terms to facilitate accurate responses. To enhance accessibility, questionnaires were translated into Lusoga and back-translated to maintain linguistic precision. For participants who did not speak English, the Lusoga version was read aloud to ensure full comprehension. Immediately after each interview, the principal investigator reviewed the completed questionnaires to check for completeness and accuracy, reducing the risk of missing or inconsistent data.

In the laboratory, standardized operating protocols and calibrated equipment were adopted to maintain the consistency and accuracy of the test results. The laboratory technician received specialized training, and periodic checks were performed to verify reliability. In addition, every tenth sample was tested independently at the Lancet laboratory, providing an extra layer of quality assurance.

### Data management and analysis

Data were collected using coded interviewer-administered questionnaires and securely stored in locked file boxes. Electronic data were entered into Microsoft Excel 2019, backed up regularly, and password-protected. The dataset was then exported to STATA version 14.2 for statistical analyses.

All the statistical analyses were conducted via STATA version 14.2. Categorical variables were summarized using frequencies and percentages, whereas continuous variables were described using means and standard deviations (SDs) for normally distributed data or medians and interquartile ranges (IQRs) for skewed distributions. Statistical significance was set at p < 0.05, and 95% confidence intervals (CIs) were reported where applicable.

The incidence of adverse neonatal outcomes was calculated separately for hyperuricemic and normouricemic mothers, with corresponding 95% confidence intervals (CIs). The chi-square test was used to assess differences between the two groups, evaluating whether hyperuricemia was significantly associated with a higher occurrence of neonatal complications. The findings were visually represented via a comparative bar chart for clarity.

To assess the associations between maternal hyperuricemia and adverse neonatal outcomes, crude relative risk (cRR) estimates were computed for each category of adverse neonatal outcomes, including preterm birth, stillbirth, and low Apgar scores. Binomial regression with a log link function (STATA command: binreg, rr) was applied to estimate cRR values, 95% CIs, and p-values. These results were presented in tabular format to highlight significant associations.

To identify predictors of adverse neonatal outcomes, a Poisson regression model with robust standard errors (STATA command: poisson, robust) was employed at both bivariate and multivariate levels to determine adjusted relative risks (aRRs). Before fitting the multivariable Poisson regression model, we assessed Multicollinearity among candidate independent variables using variance inflation factors (VIFs) and pairwise correlation matrices. All covariates included in the final model had VIF values <2, indicating no evidence of problematic Multicollinearity.

Variables with p < 0.20 in bivariate analyses, as well as those considered biologically relevant (e.g., history of preterm birth, antenatal care attendance, maternal age, BMI, and parity), were included in the final multivariate model. A p-value < 0.05 was considered statistically significant in the final adjusted analysis.

### Ethical considerations

Ethical approval was obtained from the Kampala International University Research Ethics Committee (KIU-REC-2024–448) and the Uganda National Council for Science and Technology (UNCST). Administrative clearance was obtained from JRRH. Written informed consent was obtained from all participants prior to enrollment, and confidentiality was maintained throughout the study.

## Results

### Basic characteristics of the study participants

A total of 366 women were screened for eligibility. Four were excluded (three with gestational hypertension, one with multiple gestation) and ten declined participation, leaving 352 participants for final analysis. The refusal rate was therefore 2.7% (10/366). Based on screening-log data, the age and parity distributions of women who declined participation were similar to those of enrolled participants (, suggesting minimal selection bias due to non-participation.

Among these, 176 (50.0%) were hyperuricemic and 176 (50.0%) were normouricemic. Among the 352 respondents considered for this study, the majority were aged between 30 and 39 years 162 (46.0%). The mean maternal age was 30.8 ± 6.4 years.

Most participants resided in rural areas 228 (64.8%), worked in the informal sector 238 (67.6%), were married 285 (81.0%), had mid-range income levels 211 (59.9%), and had at least secondary education 171 (48.58%) (Table 2).

**Table 2:** Sociodemographic and Clinical characteristics of the Study Participants by Hyperuricemia Status (N = 352).

Characteristic	Normouricemic n = 176 (%)	Hyperuricemic n = 176 (%)	Total n (%) N = 352	p-value
**Age (years)**				
< 20	23 (13.1)	25 (14.2)	48 (13.6)	0.667
20-29	58 (32.9)	58 (32.9)	116 (32.9)	
30-39	79 (44.9)	83 (47.2)	162 (46.0)	
40+	16 (9.1)	10 (5.7)	26 (7.4)	
**Residence**				0.655
Urban	60 (34.1)	64 (36.4)	124 (35.2)	
Rural	116 (65.9)	112 (63.6)	228 (64.8)	
**Occupation**				0.488
Unemployed	31 (17.6)	40 (22.7)	71 (20.2)	
Informal sector	123 (69.9)	115 (65.3)	238 (67.6)	
Formal sector	22 (12.5)	21 (11.9)	43 (12.2)	
**Marital Status**				0.892
Single	34 (19.3)	33 (18.8)	67 (19.0)	
Married	142 (80.7)	143 (81.2)	285 (81.0)	
**Income Level** (,000 UGX)				0.989
< 200	43 (24.4)	43 (24.4)	86 (24.4)	
200-500	105 (59.7)	106 (60.2)	211 (59.9)	
> 500	28 (15.9)	27 (15.3)	55 (15.6)	
**Education Level**				0.242
No Formal	19 (10.80)	28 (15.91)	47 (13.35)	
Primary	47 (26.70)	55 (31.25)	102 (28.98)	
Secondary	91 (51.70)	80 (45.45)	171 (48.58)	
Tertiary	19 (10.80)	13 (7.39)	32 (9.09)	
**Gestational Age**				0.111
Term (≥37 weeks)	153 (86.9)	142 (80.7)	295 (83.8)	
Preterm (<37 weeks)	23 (13.1)	34 (19.3)	57 (16.2)	
**Antenatal Visits**				0.029
< 4 visits	45 (25.6)	64 (36.4)	109 (31.0)	
≥ 4 visits	131 (74.4)	112 (63.6)	243 (69.0)	
**History of Preterm** Birth				0.882
No	149 (84.7)	150 (85.2)	299 (84.9)	
Yes	27 (15.3)	26 (14.8)	53 (15.1)	
**History of Stillbirth**				0.079
No	163 (92.6)	153 (86.9)	316 (89.8)	
Yes	13 (7.4)	23 (13.1)	36 (10.2)	
**HIV Status**				0.583
Negative	161 (91.5)	158 (89.8)	319 (90.6)	
Positive	15 (8.5)	18 (10.2)	33 (9.4)	
**BMI Category**				0.103
Underweight	14 (8.0)	18 (10.2)	32 (9.1)	
Normal	111 (63.1)	107 (60.8)	218 (61.9)	
Overweight	41 (23.3)	30 (17.0)	71 (20.2)	
Obese	10 (5.7)	21 (11.9)	31 (8.8)	

### Incidence of adverse neonatal outcomes.

**Fig 1:**
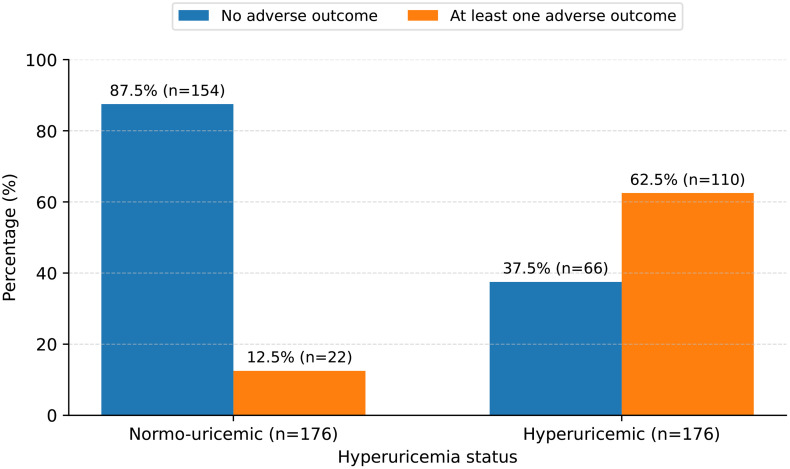
Composite incidence of adverse neonatal outcomes by hyperuricemia status (N = 176 in each group).

Overall, 132 of 352 (37.5%) participants experienced at least one adverse neonatal outcome. Preterm birth: 14.5% (51/352), SGA: 11.4% (40/352), Stillbirth: 6.8% (24/352), Low Apgar score (<7 at 5 min): 4.8% (17/352). Adverse outcomes were significantly more frequent among hyperuricemic women compared to normouricemic women (62.5% (110/176; 95% CI: 54.9–69.5) vs. 12.5% (22/176; 95% CI: 8.2–18.3), p < 0.001) (**Fig 1**).

### Incidence of specific adverse neonatal outcomes

Compared with normouricemic mothers (4.5% [8/176], 95% CI: 2.0–8.6, p < 0.001), hyperuricemic mothers had a significantly higher incidence of preterm birth (16.5% [29/176], 95% CI: 11.3–22.9). Stillbirth rates were also markedly high in hyperuricemic pregnancies (8.5% [15/176], 95% CI: 4.8–13.6) than in normouricemic pregnancies (1.7% [3/176], 95% CI: 0.4–5.0, p = 0.004). SGA was significantly more prevalent in hyperuricemic newborns (23.9% [42/176], 95% CI: 17.8–30.9) than in normouricemic newborns (8.5% [15/176], 95% CI: 4.8–13.6, p < 0.001). Low Apgar scores were comparable between the groups (24.4% [43/176] vs. 21.0% [37/176], p = 0.445), as were early neonatal deaths (1.7% vs. 4.5%, p = 0.126), indicating no significant association with hyperuricemia (**Table 3**).

**Table 3:** Association between maternal hyperuricemia and specific adverse neonatal outcomes (N = 176 in each group).

Adverse Neonatal Outcome	Exposed n = 176 (%) vs Unexposed n = 176 (%)	RR (95% CI)	p-value
Stillbirth	15 (8.5%) vs 3 (1.7%)	5.00 (1.47 - 16.99) (Ref: Normouricemic)	0.010*
Early Neonatal Death	3 (1.7%) vs 8 (4.5%)	0.38 (0.10 - 1.39) (Ref: Normouricemic)	0.143
SGA	42 (23.9%) vs 15 (8.5%)	2.80 (1.61 - 4.86) (Ref: Normouricemic)	<0.001*
Low Apgar Score	43 (24.4%) vs 37 (21.0%)	1.16 (0.79 - 1.71) (Ref: Normouricemic)	0.447
Preterm Birth	29 (16.5%) vs 8 (4.5%)	3.63 (1.70 - 7.72) (Ref: Normouricemic)	0.001*

SGA: small for gestational age, RR: relative Risk, CI: confidence interval *: statistically significant.

### Association between maternal hyperuricemia and specific adverse neonatal outcomes

The risk of stillbirth was significantly higher among hyperuricemic mothers (8.5%) than among normouricemic mothers (1.7%) (p = 0.010), with a 5-fold increased risk (RR = 5.00, 95% CI: 1.47–16.99, p = 0.010). Similarly, small for gestational age (SGA) was more common in the hyperuricemic group (23.9% vs. 8.5%, p < 0.001), with a 2.8-fold increased risk (RR = 2.80, 95% CI: 1.61–4.86, p < 0.001). Preterm birth was also significantly higher in hyperuricemic mothers (16.5% vs. 4.5%, p = 0.001), with a 3.6-fold increased risk (RR = 3.63, 95% CI: 1.70–7.72, p = 0.001). Early neonatal deaths and low Apgar scores were not significantly different between the groups (p = 0.143 and p = 0.447, respectively). Overall, maternal hyperuricemia was strongly associated with stillbirth, SGA, and preterm birth, emphasizing its role as a potential risk factor for adverse neonatal outcomes (**Table 3**).

### Additional factors associated with adverse neonatal outcomes among normotensive women at Jinja regional referral hospital

Hyperuricemic mothers were 4.32 times more likely to experience adverse neonatal outcomes, indicating that hyperuricemia is a strong predictor. Maternal age 40 + years increased the risk 1.75 times, whereas no formal or primary education increased the likelihood 2.22 and 2.31 times, respectively. Fewer than four antenatal visits increased the risk by1.32 times, emphasizing the importance of prenatal care. Preterm birth (1.38 times), prior preterm birth (1.56 times), and prior stillbirth (1.49 times) remained significant predictors of recurrent pregnancy risks. Obese mothers were 1.55 times more likely to have neonates with complications, highlighting metabolic influences. HIV status lost significance after adjustment, suggesting that other maternal factors play a greater role (**Table 4**).

**Table 4:** Additional factors associated with adverse neonatal outcomes among normotensive women at Jinja Regional Referral Hospital.

Factor	No Adverse Outcome (N = 220, %)	Adverse Outcome (N = 132, %)	cRR (95% CI)	p-value	aRR (95% CI)	p-value
**Hyperuricemia**						
Hyperuricemic	66 (30.0%)	110 (83.3%)	5.00 (3.33–7.52)	<0.001	**4.32 (2.92–6.39)**	**<0.001**
Normo-uricemic	154 (70.0%)	22 (16.7%)	1.00		1.00	
**Maternal Age (yrs)**						
<20	31 (14.1%)	17 (12.9%)	1.00		1.00	
20-29	85 (38.6%)	31 (23.5%)	0.75 (0.46–1.23)	0.257	**0.66 (0.44–0.99)**	**0.046**
30-39	93 (42.3%)	69 (52.3%)	1.20 (0.79–1.83)	0.392	1.11 (0.80–1.54)	0.529
40+	11 (5.0%)	15 (11.4%)	1.63 (0.98–2.70)	0.058	**1.75 (1.15–2.66)**	**0.009**
**Education Level**						
No Formal Education	22 (10.0%)	25 (18.9%)	2.84 (1.31–6.13)	0.008	**2.22 (1.16–4.24)**	**0.015**
Primary	49 (22.3%)	53 (40.1%)	2.77 (1.31–5.84)	0.007	**2.31 (1.23–4.35)**	**0.009**
Secondary	123 (55.9%)	48 (36.4%)	1.50 (0.69–3.20)	0.299	1.45 (0.78–2.71)	0.241
Tertiary	26 (11.8%)	6 (4.5%)	1.00		1.00	
**Antenatal Visits**						
<4 Visits	58 (26.4%)	51 (38.6%)	1.40 (1.07–1.83)	0.013	**1.32 (1.07–1.64)**	**0.011**
≥4 Visits	162 (73.6%)	81 (61.4%)	1.00		1.00	
**Gestational Age**						
Preterm	25 (11.4%)	32 (24.2%)	1.66 (1.25–2.19)	<0.001	**1.38 (1.04–1.83)**	**0.024**
Term	195 (88.6%)	100 (75.8%)	1.00		1.00	
**History of Preterm Birth**						
Yes	28 (12.7%)	25 (18.9%)	1.32 (0.95–1.82)	0.094	**1.56 (1.18–2.07)**	**0.002**
No	192 (87.3%)	107 (81.1%)	1.00		1.00	
**History of Stillbirth**						
Yes	12 (5.5%)	24 (18.2%)	1.95 (1.48–2.57)	<0.001	**1.49 (1.10–2.04)**	**0.011**
No	208 (94.5%)	108 (81.8%)	1.00		1.00	
**HIV Status**						
Positive	15 (6.8%)	18 (13.6%)	1.53 (1.08–2.16)	0.016	1.28 (0.92–1.78)	0.141
Negative	205 (93.2%)	114 (86.4%)	1.00		1.00	
**BMI Category**						
Underweight	16 (7.3%)	16 (12.1%)	1.40 (0.95–2.06)	0.093	1.31 (0.94–1.83)	0.112
Normal	140 (63.6%)	78 (59.1%)	1.00		1.00	
Overweight	53 (24.1%)	18 (13.6%)	0.71 (0.46–1.10)	0.123	0.87 (0.60–1.27)	0.470
Obese	11 (5.0%)	20 (15.2%)	1.80 (1.31–2.47)	<0.001	**1.55 (1.16–2.09)**	**0.004**

cRR: crude risk ratio, aRR: adjusted risk ratio, CI: confidence interval.

## Discussion

This prospective cohort study demonstrated that maternal hyperuricemia is a strong independent predictor of adverse neonatal outcomes, including preterm birth, small-for-gestational-age (SGA) birth, and stillbirth, even in the absence of maternal hypertension. Women with hyperuricemia had more than four-fold higher risk of a composite adverse outcome compared with normouricemic women. Additional independent predictors included advanced maternal age (≥40 years), low education, fewer than four antenatal visits, obesity, and prior adverse obstetric history. These findings complement those from a Ugandan cohort of preeclamptic women at Mbarara Regional Referral Hospital, where hyperuricemia similarly predicted adverse perinatal outcomes [15] and together they suggest that elevated uric acid may be clinically important across both hypertensive and normotensive pregnancies. The results align with those of prior studies linking elevated uric acid levels to adverse pregnancy outcomes. Amini et al. reported increased risks of preterm birth (OR = 3.17), SGA (OR = 1.28), and NICU admission (OR = 1.65) among hyperuricemic pregnancies [11]. Similarly, Daise et al. reported that low birth weight and neonatal morbidity were significantly more common in hyperuricemic mothers [18]. The underlying mechanisms likely involve placental insufficiency, oxidative stress, and endothelial dysfunction, impairing fetal growth and increasing neonatal complications [19,20]. However, discrepancies exist across studies, particularly in settings with advanced obstetric care. Laughon et al. reported that while hyperuricemia was linked to lower birth weight, it did not significantly predict NICU admission or neonatal morbidity [14]. These differences may stem from early metabolic screening, better maternal monitoring, and timely interventions in high-resource settings [21]. Additionally, variability in diagnostic criteria for hyperuricemia whether fixed cut-off or gestational age-specific thresholds may contribute to differences in reported outcomes [22].

Biologically, hyperuricemia disrupts placental function, impairing trophoblast invasion and vascularization, leading to fetal hypoxia and growth restriction [19]. Uric acid also induces the release of proinflammatory cytokines (IL-6 and TNF-α), increasing the risk of preterm labor, fetal distress, and stillbirth [23]. Additionally, high maternal uric acid levels may directly affect fetal metabolism, predisposing newborns to poor postnatal adaptation and respiratory distress [19]. These mechanisms are not restricted to preeclampsia; they may also occur in normotensive pregnancies, leading to placental insufficiency, impaired fetal growth and vulnerability to preterm birth even in the absence of elevated blood pressure.

Given the strong association between hyperuricemia and neonatal morbidity, routine uric acid screening during pregnancy may facilitate early risk identification. Interventions such as enhanced fetal monitoring, dietary modifications, and targeted pharmacologic management could mitigate neonatal risks [24]. Further research is needed to explore longitudinal uric acid trends during pregnancy and the effectiveness of intervention strategies in improving perinatal outcomes [20].

Hyperuricemia was significantly associated with stillbirth, SGA, and preterm birth, reinforcing its pathophysiological role in pregnancy complications. Stillbirth occurred in 8.5% of hyperuricemic pregnancies, a fivefold increase compared with normouricemic pregnancies (1.7%, RR = 5.00, p = 0.010). These findings support those of Daise et al, who reported that hyperuricemia was linked to increased perinatal mortality due to placental hypoxia and oxidative stress [18]. SGA incidence was nearly three times higher among hyperuricemic mothers (23.9% vs. 8.5%, RR = 2.80, p < 0.001), which is consistent with Akahori et al, who reported a strong inverse correlation between maternal uric acid and fetal growth (r = −0.59, p = 0.006) [22]. The likely mechanism involves uric acid inhibiting placental amino acid transport, reducing the fetal nutrient supply and leading to growth restriction [19].

Preterm birth was significantly common among hyperuricemic mothers (16.5% vs. 4.5%, RR = 3.63, p = 0.001). Similarly, Amini et al., reported a threefold increased risk (OR = 3.17) [11], likely due to inflammatory activation and oxidative stress triggering premature labor [20]. Our findings in normotensive women therefore support the concept that hyperuricemia may reflect broader maternal–placental vascular and inflammatory dysfunction, rather than being solely a marker of hypertensive disease

Conversely, low Apgar scores and early neonatal death did not significantly differ between the groups. These findings contrast with those of a study by Jayaraj et al., suggesting that institutional variations in neonatal care and maternal interventions may influence outcomes. [12].

Beyond hyperuricemia, several maternal characteristics were independently associated with the composite adverse neonatal outcome in this cohort. Advanced maternal age, low educational attainment, fewer than four antenatal care visits, maternal obesity, and a history of preterm birth or stillbirth all increased the likelihood of neonatal morbidity, consistent with previous work linking age-related vascular changes, social disadvantage and suboptimal antenatal care to placental dysfunction, fetal growth restriction and preterm delivery [11,14,18,20–22,24]. These factors likely act through overlapping pathways involving chronic inflammation, metabolic dysregulation and impaired uteroplacental perfusion, and may compound the adverse effects of hyperuricemia on fetal growth and perinatal adaptation [11,14,18–21,24]

In contrast, HIV status, pre-existing diabetes, renal disease and underweight BMI were not significantly associated with adverse neonatal outcomes in our adjusted models, which may reflect adequate antenatal identification and management of these conditions in this setting [23].

To our knowledge, only one Ugandan study has examined hyperuricemia in pregnancy, among women with preeclampsia in Southwestern Uganda [15]. Our prospective cohort extends this work to normotensive pregnancies and suggests that elevated maternal serum uric acid may be associated with adverse neonatal outcomes even in the absence of hypertension.

## Strengths and limitations of the study

Strengths of this study include its prospective design, gestational-age–adjusted SUA classification, and robust statistical adjustment for multiple confounders. The study was conducted in a large referral hospital, increasing generalizability to similar settings.

Limitations include a single SUA measurement, which may not reflect dynamic changes during pregnancy, and the lack of long-term neonatal follow-up. Additionally, some unmeasured confounders (e.g., dietary purine intake) could not be accounted for. Nevertheless, the strong associations observed, consistent with prior literature, support the validity of our findings.

### Conclusions

In this prospective cohort of normotensive women in Eastern Uganda, maternal hyperuricemia was associated with a significantly higher risk of preterm birth, SGA and stillbirth. These associations suggest that serum uric acid may be a useful marker of neonatal risk, but causality cannot be established from this observational study, and residual confounding is possible. Incorporating SUA testing into routine antenatal care, alongside interventions to improve ANC attendance and address modifiable risk factors, may help reduce preventable perinatal morbidity and mortality in low-resource settings.

## Supporting information

S1 File dr Hafsa excel data. (XLS)

## Abbreviations

AGA: Appropriate for Gestational Age
AOR: Adjusted Odds Ratio
AS-1: One-minute Apgar Score
AS-5: Five-minute Apgar Score
BW: Birth Weight
CDC: The Centers for Disease Control and Prevention
GFR: Glomerular Filtration Rate
HOMA: Homeostasis Model Assessment
IVH: Intraventricular Hemorrhage
JRRH: Jinja Regional Referral Hospital
LBW: Low Birth Weight
mTOR: mammalian target of rapamycin
MUA: Maternal Uric Acid
NICU: Neonatal Intensive Care Unit
NUA: Neonatal Uric Acid
PTB: Preterm Birth
RDS: Respiratory Distress Syndrome
SGA: Small for Gestational Age
SOGC: Society of Obstetricians and Gynecologists of Canada
WHO: World Health Organisation.
